# An additional k-means clustering step improves the biological features of WGCNA gene co-expression networks

**DOI:** 10.1186/s12918-017-0420-6

**Published:** 2017-04-12

**Authors:** Juan A. Botía, Jana Vandrovcova, Paola Forabosco, Sebastian Guelfi, Karishma D’Sa, The United Kingdom Brain Expression Consortium, John Hardy, Cathryn M. Lewis, Mina Ryten, Michael E. Weale

**Affiliations:** 1 Department of Molecular Neuroscience, Institute of Neurology, University College London, Queen Square, London, WC1N UK; 2 Department of Medical & Molecular Genetics, School of Medical Sciences, King’s College London, Guy’s Hospital, London, SE1 9RT UK; 3 Istituto di Ricerca Genetica e Biomedica, CNR, Cittadella Universitaria di Monserrato, Monserrato, 09042 CA Italy

**Keywords:** Gene co-expression networks on brain, K-means applied to WGCNA, Assessment of better gene clusters on bulk tissue

## Abstract

**Background:**

Weighted Gene Co-expression Network Analysis (WGCNA) is a widely used R software package for the generation of gene co-expression networks (GCN). WGCNA generates both a GCN and a derived partitioning of clusters of genes (modules). We propose k-means clustering as an additional processing step to conventional WGCNA, which we have implemented in the R package km2gcn (k-means to gene co-expression network, https://github.com/juanbot/km2gcn).

**Results:**

We assessed our method on networks created from UKBEC data (10 different human brain tissues), on networks created from GTEx data (42 human tissues, including 13 brain tissues), and on simulated networks derived from GTEx data. We observed substantially improved module properties, including: (1) few or zero misplaced genes; (2) increased counts of replicable clusters in alternate tissues (x3.1 on average); (3) improved enrichment of Gene Ontology terms (seen in 48/52 GCNs) (4) improved cell type enrichment signals (seen in 21/23 brain GCNs); and (5) more accurate partitions in simulated data according to a range of similarity indices.

**Conclusions:**

The results obtained from our investigations indicate that our k-means method, applied as an adjunct to standard WGCNA, results in better network partitions. These improved partitions enable more fruitful downstream analyses, as gene modules are more biologically meaningful.

**Electronic supplementary material:**

The online version of this article (doi:10.1186/s12918-017-0420-6) contains supplementary material, which is available to authorized users.

## Background

Systems biology is a descriptive paradigm in which one of the main concerns is how genes work together to form subsystems. A basic assumption within this context is that genes which are co-expressed are often in the same subsystem [1]. Gene co-expression networks (GCN) are graph-based models used to express such subsystems. Construction of these networks is usually based on co-variation in expression within groups of genes across samples [2]. They are graphs in which nodes are genes and edges represent interactions between them. Typically, the edges are undirected, in the sense that causality (e.g. whether changes in Gene A expression causes changes in Gene B expression) is unassigned. Edges may be weighted and/or signed, thus indicating the strength of relationship between pairs of genes and up/down regulated interactions depending on the sign. Topological considerations, such as the number or relevance of connections for each node, can distinguish some nodes as highly interconnected (hubs) and central nodes within the system being modelled.

GCN can be used to make in silico functional predictions about genes. The Guilt By Association (GBA) paradigm [3] is used to predict function for genes that are not sufficiently studied and annotated using GCNs. GBA assumes that genes that strongly co-express must share functionality, thus we can use well-characterised genes to assign function to those that are not.

Groups of genes that tightly co-express are usually seen as a single functional unit. On this basis, in the same way that single genes are used in association mapping with phenotype, convenient mathematical representations of groups of genes can be useful for multi-gene association mapping with phenotype as well. One of the most widely used pipelines for GCN construction is Weighted Gene Co-expression Network Analysis (WGCNA) [4–6]. It works in two main steps. In the first step it constructs a network *N* of gene-gene co-expression in the form of a squared *n*×*n* matrix, where *n* is the number of genes in the study and each *N*(*i*,*j*) is the interaction strength between the corresponding pair of genes (i.e. adjacency). In the second step, this matrix is used as the basis for obtaining a new squared distance matrix with the distance between genes, ready to be used for obtaining clusters. And then such clusters can be used for multi-gene association mapping with traits or different downstream analyses [7–9]. This pipeline has been widely used and generated many fruitful insights into how genes interact within specific conditions [7–13].

In this paper we propose an improvement to the standard WGCNA pipeline by a refinement of how the clusters (i.e. modules) are generated. This refinement is enabled through a hybrid clustering algorithm. It uses the output of the conventional WGCNA clustering as subsequent input to a k-means [14] clustering algorithm for further refinement. We will show that this hybrid scheme improves many interesting module properties paving the way to more accurate and potentially useful WGCNA co-expression network analyses.

WGCNA’s standard configuration uses hierarchical clustering (HC). In HC, a strong point is that the dendrogram structure eases the problem of finding a good number of clusters, *k*. Moreover, the developers of WGCNA include in the software an automated method to generate the appropriate number of clusters [15]. On the other hand, a weak point of HC is that final results strongly depend upon how distances between clusters are compared. Furthermore, once the decision on which branch of the dendrogram a gene belongs to, this cannot be undone.

Regarding k-means, a weakness in it is that the value of *k* (i.e. number of clusters) must be set prior to running the algorithm. Although there are techniques for setting it automatically, most of these are based on multiple random initialisations of centroids (e.g. k-means++ [16]), so *k* is usually set arbitrarily. It needs an initialisation of the centroids to start running. A centroid is defined as an average representative of all the genes/points within the cluster such that all genes/points belonging to the cluster show minimum distance to that centroid in comparison to the other modules. How we initialize these centroid will have a critical effect on the final result. On the upside, k-means will search for the best centroids quickly and will quickly converge to an equilibrium situation (see “Improvement of hierarchical clustering with k-means” section).

The hybrid scheme we propose exploits the upsides from both approaches while alleviating their respective drawbacks. K-means will move genes between modules thus effectively undoing premature decisions made by HC when assigning genes to sub-dendrograms. We set the value of *k* equal to the number of modules discovered by HC and we initialise the centroids to the eigengenes generated by WGCNA, thus taking advantage of HC to carry out sensible initialization (see “The standard WGCNA procedure” section).

## Implementation

### The standard WGCNA procedure

Consider a gene expression profile matrix *G*_*n*×*m*_ where n is the number of samples for a given condition, *m* is the number of transcripts and each *g*(*i*,*j*) in *G* gives the quantification of the *j*-th transcript within the *i*-th sample. The standard WGCNA [6] procedure generates a squared adjacency matrix, between genes, based on their correlation. Depending on whether the adjacency is signed (where correlations in the [−1,1] interval are scaled into the [0,1] interval) or unsigned (where negative correlations are made positive) we will obtain networks either reflecting the direction of co-regulation (i.e. up or down regulation) or ignoring it, respectively. Adjacency is defined as *a* *d* *j*(*i*,*j*)=|*c* *o* *r*(*i*,*j*)|^*β*^ for genes *i* and *j*. The *β* parameter is an integer that modulates how smooth is the transition between the lowest to the highest possible co-regulation between genes.

The WGCNA methodology enables choosing *β* in such a way that the network shows a Scale Free Topology (SFT) property [17] (where the network has the same shape whether ‘zoomed-out’ or ‘zoomed-in’). This feature is commonly observed in biological networks. From the adjacency values, a new matrix with the same dimensions is created, the Topological Overlap Matrix (TOM). This step alleviates the effect of noisy genes when obtaining the adjacency from correlation.

Once the network is built through the TOM, it is converted to a distance matrix (1−*T* *O* *M*) to use it as the basis for clustering (HM with average linkage distance comparison between clusters). A dynamic tree-cutting algorithm [15] is then applied to the dendrogram to generate a partition *P*={*P*_1_,…,*P*_*k*_} of disjunct sets of genes.

Thus, WGCNA generates two main components which are useful for subsequent downstream analyses. On the one hand, the TOM gives, for the *j*-th row/column, the level of co-expression of gene *j* with all of the genes in the network. The higher the value for a given (*i*,*j*) pair, the tighter the interaction between them. Furthermore, the sum of all row or column values for a gene, will give a measure of its overall level of co-regulation within the experimental condition, i.e. its ‘hubbiness’. Thus, the TOM is, in effect, the GCN.

The other component produced by WGCNA is the partition of gene sets, *P*, created from the TOM. These partitions or modules often reflect cell types, common cellular functions or other biological subsystems reflecting, for example, immune function, or function related to the tissue under study [2, 7, 8]. But the main utility of modules is to allow mapping gene groups to traits, when available. Following the WGCNA standard methodology, this is performed by looking for significant correlations between traits and the module ‘eigengene’. The eigengene summarizes the overall module activity in a given sample, and is obtained as the 1st PC component of the gene expression of genes belonging to the module.

### Improvement of hierarchical clustering with k-means

HC provides a convenient graphical representation of groupings that can be validated by biologists. One can readily obtain a suitable number of clusters from such an approach by ‘cutting’ the dendrogram at different heights, either manually or via various automatic algorithms [15]. But, as we explained above, the final dendrogram strongly depends on how we measure distance between clusters (e.g. via simple, complete or average linkage). Furthermore, once a gene falls under a subdendrogram, this decision cannot be modified under HC.

If we consider how WGCNA manages modules and eigengenes, it is assumed that each gene is highly correlated with other genes in its module. In other words, the module membership (MM) of the gene in its own module, measured as the Pearson correlation between its expression and the module eigengene, should be higher than it is for any other module. However, we show here, from our real-data analyses, that 25% of genes would be better off in other modules (see “K-means improves the ‘eigengene’ as a tool for analysis” section).

In this paper, we propose a post-processing step based on k-means to overcome all these limitations. It works on the partition *P*, leaving the TOM unmodified. The k-means algorithm [14] is well known and works on the *n* dimensional sample space of *m* points (genes) in an iterative fashion. It starts by setting a value for *k*, the number of clusters to discover and *k* centroids, one for each cluster. Centroids are the representatives of each cluster, in such a way that a point (gene) *g* belongs to cluster *i* if the distance of such point to the cluster centroid is the minimum among all distances to all *k* cluster centroids. In standard k-means, given a partition of *k* modules, the the centroid for the *i*-th module *c*_*i*_={*c*_*i*,1_,…,*c*_*i*,*n*_} is generated as follows

1 $$ c_{i}=\frac{1}{n}\sum\limits_{j=1}^{m} {g_{j}},\text{where}~g_{i}\in p_{i}.   $$

However, in WGCNA, the notion of a centroid is substituted by that of an eigengene. Accordingly, our definition of k-means will use eigengenes as centroids.

The concept of distance is a central element of k-means. It is important to note that distance in k-means is always defined between a point in the dataset (i.e. a gene) and a centroid (i.e. an eigengene). Euclidean distance is the the most commonly used distance in conventional k-means. However, given that we are constructing co-expression networks based on correlations, distance cannot be Euclidean. Modules should represent co-expressed genes (i.e. highly correlated) instead. Thus, and depending on the WGCNA type of network, we should apply a distance between gene and eigengene based on the co-expression measure used. We will limit our discussion to signed networks. These specific types of networks will separate up- from down-regulated genes in different modules, which is usually of biological interest. They are also convenient for downstream analysis as correlation of genes and eigengenes will be positive, which eases a posteriori analyses. In signed networks, WGCNA uses

2 $$ co(g_{i},eg_{j})=\frac{1}{2}(1+cor(g_{i},eg_{j})),  $$

as a normalised measure of co-expression between the expression profile of a gene *g*_*i*_ and a eigengene *e* *g*_*j*_, where by default *c* *o* *r*() is the Pearson correlation coefficient. Accordingly, we use 1−*c* *o*(*g*_*i*_,*e* *g*_*j*_) as distance. It is worth noting that HC needs a distance matrix between all genes, i.e. 1−*T* *O* *M*. K-means needs instead a computable distance definition between gene and eigengene. Finally, on the basis of this definition of centroid and distance, genes are reassigned to the partitions induced by the new centroids, iteratively. If a stopping criterion is met, the algorithm finishes. Otherwise, a new iteration is performed.

We note that WGCNA is computationally optimized to use Pearson correlation. Other correlation measures are in principle possible, including Spearman’s rank correlation coefficient. However, in our own investigations we observe an increase in computation time of at least ×2.5 when using Spearman correlation without seeing any conclusive improvement with respect to the biology of networks (data not shown). Thus, throughout this paper we perform analyses using Pearson correlation.

We propose a general procedure which obtains, from a *G*_*n*×*m*_ matrix of gene expression profiles from *n* samples and *m* genes, a clustering partition *P* of such genes by incorporating the standard WGCNA process together with a post-processing of the partition obtained from it. The original contribution of this paper is described in steps from 5 to 8 below.

- Step 1: Initialization. Let *G*_*n*×*m*_ be a dataset of *n* samples and *m* genes for a given condition. Let *d*() denote a distance function between a gene in *G* and an eigengene. Let *f*_*c*_() denote a function which takes a clustering partition *P*={*p*_1_,…,*p*_*k*_} as an argument and generates centroids (i.e. *k* vectors, one for each *p*_*i*_, of *n* components)
- Step 2: *β*=WGCNA::pickSoftThreshold(data=G, powerVector=1:20, networkType=‘signed’)$powerEstimate
- Step 3: Obtain a *TOM*, given *G* and *β*
- Step 4: Generate a partition *P*_*HC*_={*p*_1_,…,*p*_*k*_} with 1−*T* *O* *M* as a distance matrix and with average linkage hierarchical clustering and dynamic cutting height.
- Step 5: Let *c*={*c*_1_,…,*c*_*k*_} be a set of *k* vectors of *n* components which denote the centroids of the k-means clustering.
- Step 6: Initialize *c* with *f*_*c*_(*P*_*HC*_)
- Step 7: Create a new partition *P*_*kM*_={*p*_1_,…,*p*_*k*_} by assigning each gene *g*_*i*_, 1≤*i*≤*m* to a *p*_*j*_∈*P*_*kM*_ such that *d*(*g*_*i*_,*c*_*j*_)≤*d*(*g*_*i*_,*c*_*t*_), 1≤*t*≤*k* holds.
- Step 8: If the termination criterion holds, then STOP. If not, generate new centroids *c* with *f*_*c*_(*P*_*kM*_) and Go to Step 7.

Note that, in this algorithm, we left *d*() and *f*_*c*_() undefined. However, within this paper we define *d*() according to Eq. 2 and *f*_*c*_() as the module eigengene.

#### Computational complexity of the proposed approach

Conventional WGCNA GCN construction needs three sequential steps: (1) obtaining the soft threshold (i.e. *β* parameter) to account for scale free topology, which has a computational complexity that depends on the number of genes and samples, (2) obtaining the TOM matrix, which has a complexity *O*(*n* ^2^), where *n* is the number of genes, as it has to construct a *n*×*n* squared matrix of adjacencies between all *n* genes, and (3) hierarchical clustering, which has a complexity *O*(*n* *l* *o* *g* *n*). Overall, WGCNA’s computational complexity is *O*(*n* ^2^). WGNCA’s space complexity is also *O*(*n* ^2^) because it needs to maintain the TOM in memory for HC to get the clusters. The computational complexity of k-means fits well with WGCNA’s complexity. Its time complexity is *O*(*n*×*k*×*i* *t*) where *n* is the number of genes, *k* is the number of clusters and *it* is the number of iterations. Assuming that *k*,*i* *t*<100, using it as a post-process is very affordable in terms of computation time. Note that k-means does not require the TOM matrix in memory as the only distances it requires are between genes and eigengenes, and these we obtain on the fly by using Eq. 2.

#### Stopping criterion

It is reasonable to assume that a sufficiently high number of k-means iterations will always be able to decrease the number of misplaced genes (i.e. genes which lie closer to the centroid of a different module) to 0. On the other hand, the algorithm’s time complexity (see “Computational complexity of the proposed approach” section) means that it is possible to run a single k-means in a conventional laptop in a matter of a few minutes. This means that we could, in principle, design a stopping criterion based on the minimun number of misplaced genes being set to 0. However, we note that a situation may exist where the algorithm may fall into an infinite loop without reaching the desired state (i.e. changing the same genes from one module to another and back again). Thus, the stopping criterion we include in the software package km2gcn tries to reach the desired value for misplaced genes but always within a limited number of iterations. We did not observe the mentioned *infinite loop* situation in any of our experiments.

## Results

We wished to assess the ability of our method to define gene groups that genuinely reflect biological function. This is non-trivial for the following reasons. Firstly, many genes are known to be pleiotropic, i.e. a single gene can affect many traits [18]. Transcription factors are a good example of this [19] but there are many other examples [20]. By creating non-overlapping partitions we deliberately ignore this fact and implicitly assume a model in which genes are highly specialized (i.e. belong to a single module). Secondly, we are limited by technology and sample availability from producing optimum estimates of gene expression profiles. We therefore lack of all the necessary information to build the best model. Finally, if we wanted to evaluate the functional similarity of genes within a module, again we do not know all functions that all genes may play in any condition.

Notwithstanding these caveats, we explored various approaches to provide a comprehensive and varied assessment of the effectiveness of our k-means hybrid method. In “Materials and methods for the GCNs used for our evaluations” section we describe the datasets used in our investigations and the particular pipelines used to obtain the corresponding GNCs. In “Dynamics of k-means when working on 1-TOM distance space” section we show our hybrid approach (i.e. the combination of HC and k-means) works. in “Is k-means doing a proper job?” section we digress to note that k-means actually optimizes the sum of squares of within cluster distance. In “K-means improves the ‘eigengene’ as a tool for analysis” section we show that the proposed approach improves modules as a tool for mapping with traits. In “K-means improves module preservation” section we suggest that k-means improves cluster similarity between conditions (i.e. tissues in this case). In “K-means detects more accurate partitions than WGCNA in simulated data” section we compare the accuracy of k-means against WGCNA on simulated data. In “K-means improves functional enrichments” section we show that k-means improves a module’s functional characterization through well-known databases such as the Gene Ontology. Finally, in “K-means improves brain specific cell type marker enrichment” section we present results that suggest that gene markers for specific cell types show a better arrangement in partitions generated from k-means.

### Materials and methods for the GCNs used for our evaluations

We evaluated GCNs in two well-known datasets. The first (the United Kingdom Brain Expression Consortium or UKBEC dataset) is focused on brain tissue exclusively and it is based on Affymetrix Human Exon v2 microarray expression profiles from 10 brain tissues. This dataset is well suited for evaluating the k-means extension to WGCNA because it is well known, it comprises 10 different brain regions and GCN networks created with the standard WGCNA method have been published [8]. The procedure used to create the GCNs is as follows. Sample outliers were identified by visual inspection after clustering the samples using hierarchical clustering with Euclidean distance as the distance measure. The majority of the identified outliers had low interarray correlation, which is defined as the Pearson correlation coefficient of the expression levels for a given pair of transcripts using all available data available (i.e., <3 standard deviations of the average interarray correlation). After outlier removal, the same process was repeated to check for additional outliers. The GCN constructed was of signed type, with *β*=12 for all tissues. Using these settings, the HC WGCNA partition was created using 15,409 transcripts (13,706 genes) passing quality control. Once the partition was created, 3743 additional transcripts (3541 genes) were assigned to modules based on their highest module membership. Each partition was refined afterwards with k-means.

The second dataset is GTEx [21], which is one of most comprehensive human datasets currently available for multi-tissue transcriptomics. The GTEx V6 gene expression dataset comprises 11,978 samples unevenly distributed across 54 post-morten human tissues. We created networks for 42 tissues. In sequential steps, starting from RPKM [22] values of gene-level quantification provided by GTEx, we selected all tissues with more than 60 samples. For each tissue we retained Ensembl genes with *R* *P* *K* *M*>0.1 seen in more than 80% of the samples. This produced a variable set of genes for each tissue, with a minimum of 16,098 for skeletal muscle tissue and a maximum of 29,561 for testis. We applied batch, gender, age and RIN as known covariates for data correction and to account for unknown covariates we applied SVA (surrogate variable analysis)[23] axes. For each dataset of filtered RPKM values, we applied the sva R package using svaseq() to generate SVA axes. For network construction we used the residuals obtained by regressing the RPKM expression values with the known and unknown covariates with a generalized linear model. To construct the networks, we applied the algorithm introduced in “Improvement of hierarchical clustering with k-means” section.

Note that the differences between the UKBEC and GTEx networks are important and makes them well suited and complementary for the purpose of our study. The UKBEC gene expression dataset is microarray based, while the GTEx gene expression dataset is based on RNA-seq technology, with RPKM quantification. UKBEC networks are restricted to 10 brain tissues while GTEx networks cover 42 tissues, including 13 brain tissues (see Additional file 1 for tissues used, number of samples and genes). In summary, we have 42 GTEx GNCs, 10 brain specific UKBEC GCNs, GTEx sample sizes range from *n*=63 to *n*=430 (mean 182); UKBEC sample sizes range from *n*=65 to *n*=88 (mean 78.8); we have a variable number of genes used in the GTEx GCNs, in the range 16,098 to 29,561 for skeletal muscle and testis respectively (mean 19,636); and the same 19,152 probes for all 10 UKBEC GCNs. Finally, note there is a much higher variability in the number of modules per GCN in GTEx, [10,214] (mean 67.6) than in UKBEC, [13,34] (mean 22).

Please note that throught the paper we use abbreviations to refer to tissues. Please see Table 1 for the correspondence between abbreviations and brain region names.

**Table 1:** Real names for the tissues used in the UKBEC and GTEx brain tissue experiments

Short name	UKBEC Tissue name	Samples	Short name	GTEx Tissue name	Samples
CRBL	Cerebellum	76	AMYG	Amygdala	72
FCTX	Frontal Cortex	83	ACCT	Anterior cingulate cortex (BA24)	84
HIPP	Hippocampus	86	CAUD	Caudate (basal ganglia)	117
MEDU	Medulla	88	CEHE	Cerebellar Hemisphere	105
OCTX	Occipital cortex	77	CERE	Cerebellum	125
PUTM	Putamen	77	CTEX	Cortex	114
SNIG	Substantia nigra	65	FCTX	Frontal Cortex (BA9)	108
TCTX	Temporal Cortex	72	HIPP	Hippocampus	94
THAL	Thalamus	81	HYPO	Hyppothalamus	96
WHMT	White matter	83	NUAC	Nucleus accumbens (basal ganglia)	113
			PUTM	Putamen	97
			SPIN	Spinal Cord	71
			SNIG	Substantianigra	63

### Dynamics of k-means when working on 1-TOM distance space

As outlined in “Improvement of hierarchical clustering with k-means” section, our proposed algorithm does not modify the distance matrix (i.e. 1−*T* *O* *M*) but acts later, on the partition *P*={*p*_1_,…,*p*_*k*_} taking *k* from the number of modules discovered by the HC used within WGCNA. K-means acts iteratively creating centroids from modules and deciding for each gene, on the basis of the new centroids, which one is nearest to the gene. If, in the current iteration, the gene is nearest to a different centroid, then the k-means algorithm assigns it to the corresponding module. Thus, in each iteration a new partition is generated with the changes applied to the former partition.

**Fig. 1:**
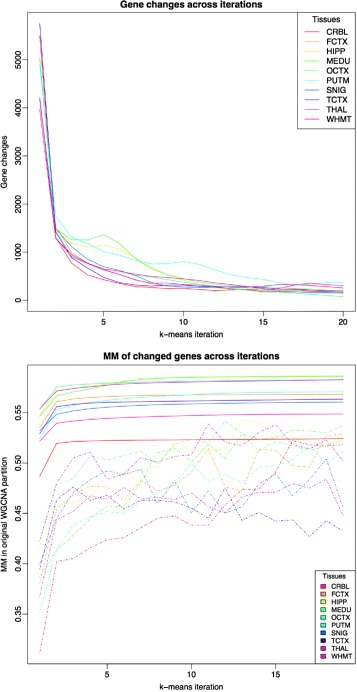
*Upper plot* shows the evolution of the number of moved genes (*y axis*) between any pair of modules *p*_*i*_ and *p*_*j*_ across k-means iterations (*x axis*) for UKBEC-microarray dataset. *Bottom plot* shows the average module membership of genes (*y axis*) moved (*dashed line*) across iterations (*x axis*) for the UKBEC-microarray dataset in comparison with average module membership for all the genes (*solid line*)

Figure 1 displays the dynamics of the algorithm in terms of how genes are changed from one module to another. In all analyses displayed there is a high activity in terms of moved genes in the early iterations, which progressively decreases to reach a stable level of changes close to zero. The number of changes at the first iteration ranges roughly between 3000 and 5000 genes, i.e. about 1/4 of the gene pool size. Any single gene can be moved more than once during the series of iterations (for more details on gene changes and how the algorithm stabilizes see “Is k-means doing a proper job?” section). It is also of interest to note that multiple modules contribute to the final configuration of genes to each other module. For example, with the 42 GTEx GCNs, for each module *p*_*i*_∈*P*, on average 30% of other modules within the GCN contribute with genes to its final gene set configuration. Genes that leave their HC module have a module membership at that module of 0.53 on average, with standard deviation of 0.19. Genes arriving to a module for the final k-means partition show an average MM on arrival, 0.57, with standard deviation 0.18.

The lower panel of Fig. 1 focuses on the 10 UKBEC GCNs, and on how MM evolves with iterations. Dashed lines show, for each tissue, the average MM of moved genes, at each iteration, defining MM with reference to the original WGCNA partition. Initially, the algorithm focuses on moving genes with very low MM, but following this it focuses on genes with higher MM and then stabilizes. The solid lines show the average MM of all the genes in the network for each iteration. This dramatically increases over the first iterations and then smoothly and monotonically increases across additional iterations. This suggests that, over time, genes’ assignment to a module becomes stronger.

### Is k-means doing a proper job?

k-means is designed to optimize the sum of squares of within clusters distance [14]. We define the within cluster distance, denoted with *W*(*P*), for a partition *P* as

3 $$ W(P)=\sum\limits_{k=1}^{K}\sum_{p(i)=k}||g_{i}-c_{k}||^{2}  $$

where *K* is the number of clusters within *P*, *p*(*i*) refers to the cluster that *g*_*i*_ belongs to, and *c*_*k*_ is the eigengene for the *k*-th cluster. We could, alternatively, define the distance between clusters, *B*(*P*). Note that for a given set of genes, we can obtain the sum of distances between all gene pairs. If we denote this measure by *T*(*G*) for a given gene expression profile *G*, we can decompose it into *T*(*G*)=*B*(*P*)+*W*(*P*) for any given *P* obtained from *G* (see [24] for a detailed discussion). This means that maximizing the between-clusters distance is equivalent to minimizing the within-cluster distance.

**Fig. 2:**
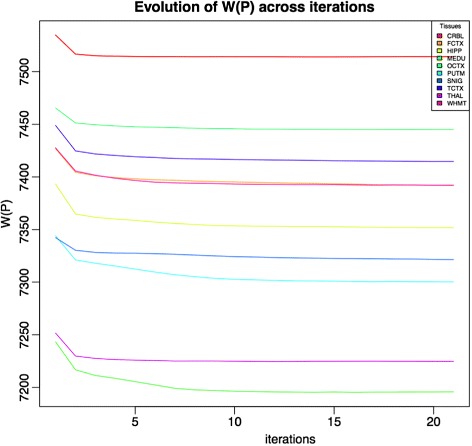
The within cluster distance evolution during the k-means runs for the UKBEC datasets

We observe that k-means monotonically decreases *W*(*P*) (and thus *B*(*P*) increases) across iterations (see Fig. 2). The algorithm generates a higher *W*(*P*) at the early iterations, which decreases to a lower level in later iterations. This behaviour is in line with the shape of the gene changes curves of the upper plot in Fig. 1. Higher number of moved genes imply higher decreasing rate of within cluster distance.

**Fig. 3:**
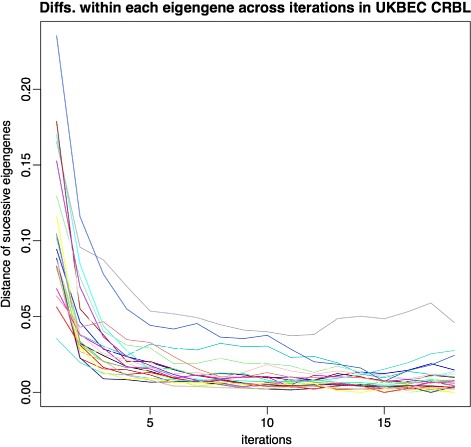
Euclidean distance of successive module eigengenes along the k-means iterations for Cerebellum samples for UKBEC datasets

This behaviour is also in accordance with what we see in Fig. 3. This plot shows, for each module *p*_*i*_∈*P*, and the specific case of UKBEC’s cerebellum CGN, the distance between the eigengenes for the same module, as they are created during successive iterations. Over time, the eigengene vectors stabilize across iterations suggesting that cluster definition becomes stable.

### K-means improves the ‘eigengene’ as a tool for analysis

One of the main applications of WGCNA partitions is searching for associations between gene clusters and traits. Traits are usually given as a vector of *n* components where *n* is the number of samples. On the other hand, modules are comprised of *m* vectors of the same form where each vector is the expression profile for the corresponding gene. To assess the correlation between the trait and the gene module, WGCNA transforms module *p*_*i*_ into an eigengene (i.e. the 1st principal component of gene expression). From this, the correlation between the trait and the eigengene can be easily obtained.

There are several applications for the eigengene. For example, it can be used to provide a measure of how strong is the membership of each gene *g*∈*p*_*i*_ to the *i*-th module, by correlating its expression with the eigengene, resulting in the MM of *g*. Let this module membership be denoted with *m*(*g*,*i*) for gene *g* and module *i*. It is assumed that a good *P* would be one such that, the number of genes *g* with *m*(*g*,*i*)<*m*(*g*,*j*) when *g*∈*p*_*i*_, for any *i*≠*j*, is low. Let us call such genes ‘misplaced genes’. We would prefer a partition in which the number of misplaced genes is minimum. To assess the number of misplaced genes after applying k-means, we performed investigations in the 10 UKBEC and 42 GTEx tissues. In UKBEC, using k-means with only 20 iterations (i.e. the fixed number of iterations used in all the experiments), we get a maximum of 380 misplaced genes in the putamen and a minimum of 72 for occipital cortex and a mean of 208 misplaced genes per partition. In the WGCNA partitions, the maximum number of misplaced genes is 5742 in temporal cortex, the minimum is 3970 in white matter and an average of 4763 genes; 20 times more than with the k-means algorithm. In GTEx clustering partition modules, the average number of misplaced genes in modules from a WGCNA partition is 118. After applying k-means, it is only 0.4.

### K-means improves module preservation

One component of WGCNA provides a convenient tool for the analysis of module preservation [5]. Given a partition, *P*, constructed from a network obtained from a given set of samples *S*, we can test whether the features of each module *p*_*i*_∈*P* (i.e. cluster and network based features) are preserved in an alternative set of samples *S* ^′^ (e.g. a different species but same brain region, or same species but different brain region). Preservation analysis is based on estimating, for some statistic of interest, differences between what is observed and what is obtained by random permutation. For example, one statistic of interest is the gene correlation with the eigengene (kME). Through a simple transformation one can check whether the values obtained in the reference network are maintained (i.e. correlated) for the same genes within the other network. WGCNA uses the ‘Z-summary’ statistic as a general summary of all the different statistics used. To assess the effect of k-means on Z-summary, we performed the same investigations on both 10 UKBEC brain tissues and on the 13 GTEx brain tissues. Note we focus on brain tissues within GTEx as comparison of preservation only makes sense for tissues that are similar. Within each UKBEC and GTEx tissue GCN, we compared the preservation of all the partitions generated by WGCNA alone with the preservation obtained by applying k-means to each of them. A permutation analysis on 10 tissues generates, for each tissue *t*, and for each module *p*_*i*_ within the corresponding tissue network, a vector of 9 Z-summary values corresponding to the preservation of *p*_*i*_ in the other 9 tissues.

Table 2 displays the results of the comparison between WGCNA and k-means. Each table cell indicates the difference between the number of modules preserved after applying k-means, versus the number of modules preserved with standard WGCNA (defined as Z-summary > 10 following the author’s recommendation). For example, in subtable (a), FCTX (row) shows 5 more modules preserved in CRBL (column) after applying the k-means method.

**Table 2:** Number of new modules from a tissue (rows) that are preserved on another tissue (columns) after applying the k-means to the standard WGCNA partitions

(a) UKBEC brain tissues													
	CRBL	FCTX	HIPP	MEDU	OCTX	PUTM	SNIG	TCTX	THAL	WHMT			
CRBL	0	1	2	3	0	5	3	1	3	2			
FCTX	5	0	1	5	0	6	3	0	5	3			
HIPP	4	2	0	3	0	7	1	3	0	0			
MEDU	1	2	4	0	3	2	3	2	1	1			
OCTX	7	1	3	6	0	9	6	3	8	6			
PUTM	3	1	3	2	1	0	1	1	0	2			
SNIG	1	2	1	0	1	1	0	0	0	1			
TCTX	3	2	1	3	0	1	4	0	2	4			
THAL	2	2	3	1	1	1	0	0	0	-1			
WHMT	1	1	0	1	1	0	1	1	0	0			
(b) GTEx brain tissues													
	AMYG	ACCT	CAUD	CEHE	CERE	CTEX	FCTX	HIPP	HYPO	NUAC	PUTM	SPIN	SNIG
AMYG	0	0	14	1	2	5	6	3	10	14	7	7	7
ACCT	0	0	0	3	1	3	-1	5	1	0	1	6	2
CAUD	2	3	0	2	-1	5	6	5	6	3	1	2	4
CEHE	2	0	1	0	8	0	0	0	4	1	2	0	0
CERE	1	2	1	15	0	4	1	1	0	0	1	1	1
CTEX	2	4	0	4	3	0	1	2	4	4	4	6	3
FCTX	4	5	6	1	2	4	0	3	0	1	4	1	2
HIPP	0	4	7	2	4	0	8	0	1	2	2	8	0
HYPO	2	9	7	4	5	6	5	11	0	3	4	1	1
NUAC	12	11	7	9	5	9	7	20	9	0	6	4	7
PUTM	1	-3	4	6	1	5	3	8	7	5	0	6	5
SPIN	4	2	1	-1	1	6	2	7	9	5	14	0	4
SNIG	15	12	5	0	0	18	4	9	14	7	13	6	0

From Table 2 it is apparent that there is an overall increase in the number of modules preserved under k-means. In the UKBEC GNCs, there is an improvement in 73 cases (81%), no improvement in 16 cases (17%), and only case with a worse preservation (thalamus in white matter). The average improvement in modules preserved for UKBEC is 2.1. In the GTEx GCNs, there is an improvement in module preservation in 133 cases (85%), no improvement in only 20 cases (12.8%) and a decreased preservation in just 3 cases. The average number of modules improved by the k-means method is 4.2 (note that in GTEx networks we get higher number of modules per GCN).

This suggests that k-means creates less noisy modules as similarities between tissues are more apparent. Finally, it is worth noting that each tissue is expected to have specific modules, i.e. modules that will be poorly preserved in other tissues because they are exclusive from that tissue, reflecting study-specific or sample-specific gene subsystems.

### K-means detects more accurate partitions than WGCNA in simulated data

We wanted to test whether k-means improves the accuracy of partitions *P* with respect to those obtained under standard WGCNA. To this end we investigated networks based on ‘synthetic data’. The WGCNA package provides a gene expression profiling simulation method simulateDatExp(), which is a convenient method for generating artificial data sets that mimic the properties of real datasets. The simulation method works with the eigengene of gene expression for each gene belonging to the module.

The simulation method requires, as arguments: (1) a matrix with the eigengene for each module; (2) the proportion of the total gene pool that one will find within each module; and (3) the number of genes to be simulated. Note that the number of samples we want to simulate appears implicitly as the length of each eigengene (each eigengene has as many components as samples used to construct the GCN). The method returns two elements: (1) a gene expression profiling data set, let us denote it with *D*, that we can use to construct GCNs; and (2) the ideal clustering partition of the simulated gene expression profiling, here denoted by *P*(*D*). Thus, if we rely on the effectiveness of this simulation method, then a simulated data set *D*, we will prefer a GCN construction algorithm *A* to algorithm *B* if the distance between *A*(*D*) and *P*(*D*) is smaller than between *B*(*D*) and *P*(*D*), where *A*(*D*) and *B*(*D*) are the clustering partitions we get after constructing GCNs on *D* with *A* and *B*, respectively. The accuracy of an algorithm *A* is defined by the similarity of the theoretical optimal partition within the synthetic data to the partition constructed by *A*.

In order to test whether k-means performs any better than standard WGCNA on simulated data, we constructed a plausible set of simulated gene expression profiles. We used GTEx and test with them both k-means and standard WGCNA on GCN construction.

The accuracy of an algorithm *A* will be defined as how similar are the theoretical optimal partition within the synthetic data, and the partition constructed by *A*.

In order to test whether k-means performs any better than standard WGCNA on simulated data, we constructed a plausible set of simulated gene expression profiles. We used GTEx standard WGCNA GCNs (i.e. their eigengenes and module relative size) as the simulation seed for the generation of a synthetic gene expression profile. We focused on the GTEx dataset rather than UKBEC, because the 42 GCNs comprise a usefully heterogeneous network dataset. The simulated data process produced a gene expression profile and a theoretical ideal clustering partition for such profile. We used this theoretical ideal partition to evaluate standard WGCNA and k-means accuracy. To estimate accuracy we use three different statistics: (1) the Rand [25] index, also implemented within WGCNA, the Jaccard coefficient and the similarity index [26], all of them implemented within clv R package.

Results for all the experiments appear in Additional file 2. Each row corresponds to a GTEx tissue, the randsimvswgcna column corresponds to the Rand index between the ideal partition and that obtained with WGCNA on the simulated data. The randsimvskm column corresponds to the same index when using k-means. The other four columns correspond to the Jaccard coefficient and the similarity index.

**Fig. 4:**
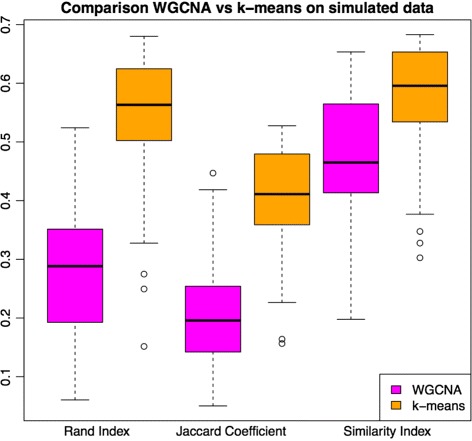
Results on performance of standard WGCNA and k-means on 42 simulated data sets that used the GTEx WGCNA GNCs as seed for simulation. We display the same results using three different indexes of similarity between cluster partitions. The k-means method outperforms standard WGCNA with all three indexes used

The k-means refinement generate higher values in all the cases for all three indexes. These results are illustrated in Fig. 4.

### K-means improves functional enrichments

The Gene Ontology [27] is a curated database for gene annotation which can be used for the functional characterization of gene sets. Given a set of genes (i.e. the gene set used to create our GCN), and a subset of those genes (i.e. a module within our partition *P*), an enrichment analysis can be performed on GO annotations [28] to search for terms in the ontology that are significantly enriched in the subset of genes relative to the full set. The number and strength of significant terms obtained in this way can be used to measure the biological functionality of the module.

Given two different partitions *P* and *P* ^′^ created from the same TOM, we would prefer the partition that generates more significant GO terms if we assume GO to reflect a biological ground truth as this would suggest that the preferred partition makes more biological sense. We used the gProfiler R package [29] to obtain enrichment *p*-values, avoiding EIA (Electronic Inferred Annotations) terms in GO and requiring a correction for multiple testing with gSCS, as developed by the authors of the package. We describe below a series of investigations to characterize the improvement in a module’s biological functionality.

#### Global annotation term significance

Consider a partition *P*={*p*_1_,…,*p*_*k*_} of genes arranged into modules *p*_*i*_, 1≤*i*≤*k*. Now suppose we want to perform a gene set enrichment analysis on each *p*_*i*_∈*P* based on the Gene Ontology. GO is a list of ontological terms, organised into three main branches: BP (Biological Process), MF (Molecular Function) and CC (Cellular component). Genes within the database will be associated with a number of terms from each branch. Thus, for each term in GO, and given the list of genes in *p*_*i*_, we can apply a contingency test, e.g. Fisher exact test [30], under the null hypothesis that the genes in *p*_*i*_ show no significant overlap with the set of genes associated with the term. With an appropriate correction for multiple testing, we define as significant the association of the list of genes in *p*_*i*_ with the corresponding term, when the corrected *p*-value is <0.05. We then aggregate all these *p*-values for a module in a single measure of significance as follows. For each *p*_*i*_∈*P*, we use

4 $$ s_{GO}(p_{i})=\sum\limits_{pvalue_{j}\in test(p_{i},GO)}{-log_{10}(pvalue_{j})},  $$

where *t* *e* *s* *t*(*p*_*i*_,*G* *O*) is the set of *p*-values, *p* *v* *a* *l* *u* *e*_*j*_, of significant terms associated with the genes in partition *p*_*i*_, emerging from the analysis. In this way,

5 $$ S_{GO}(P)=\sum\limits_{p_{i}\in P} s_{GO}(p_{i})  $$

can be used to aggregate all the biological signals (i.e. all the significant annotation terms) of a whole partition *P*. Given a choice of partitions, we prefer *P* to *P* ^′^ when *S*_*GO*_(*P*)>*S*_*GO*_(*P* ^′^).

**Fig. 5:**
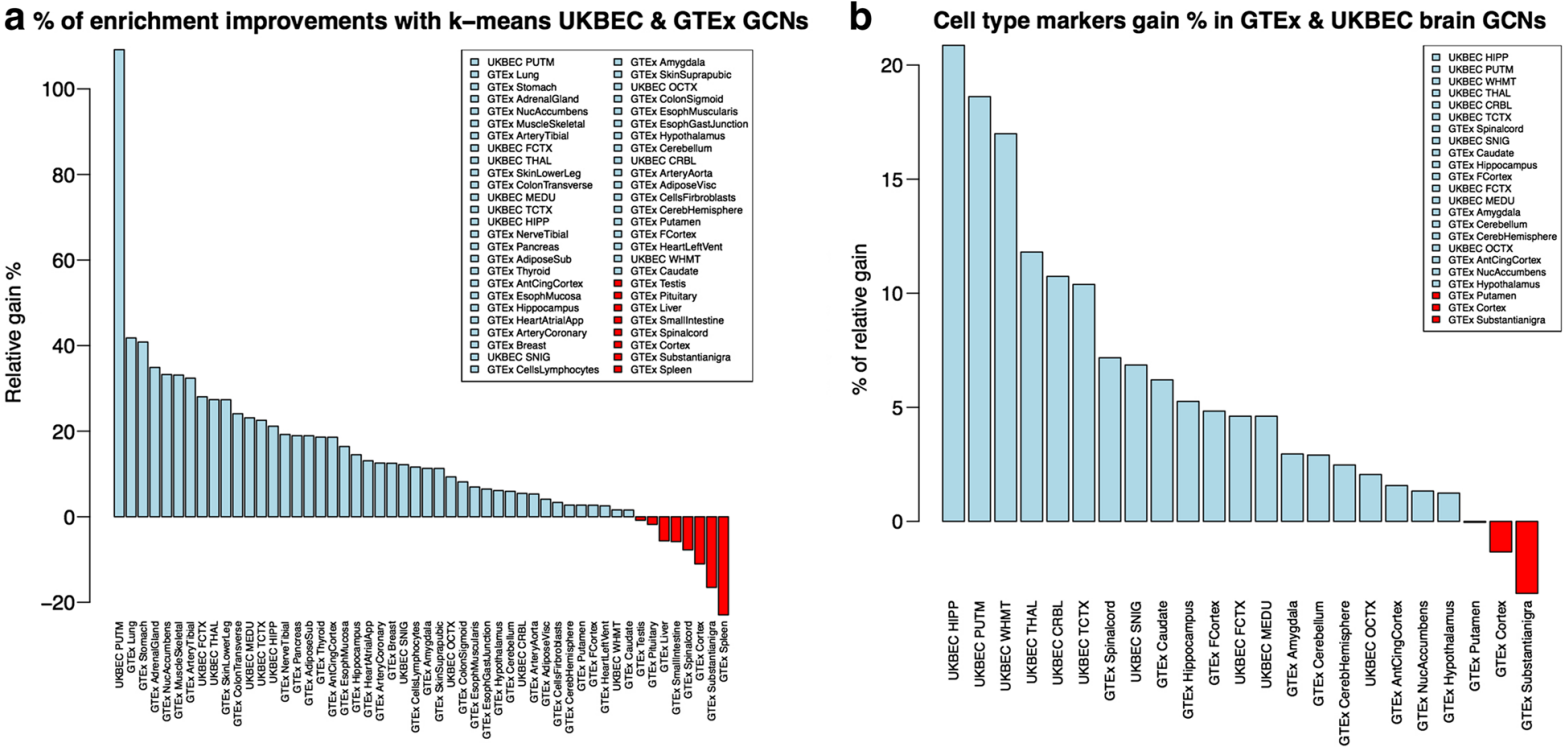
The *left plot’s light blue blue bars* show the percentage of relative improvement by k-means with respect to WGCNA *S*_*GO*_(*P*) statistic. Values in *red* (<0%) are those that k-means fails to improve. The right plot shows cell type enrichment improvement in the same way, for the 10 UKBEC GCNs and the 13 GTEx brain networks. Again, values in *red* are those that k-means fails to improve

Figure 5 a displays for each UKBEC and GTEx GCN, the relative improvement between the standard WGCNA partition, *P* and the k-means partition, *P* ^′^ by

$$\frac{S_{GO}(P')}{S_{GO}(P)} - 1. $$

The average improvement is 13% (ranging from -22.9% for the GTEx Spleen GCN to 109.1% for the UKBEC Putamen GCN). Overall, there is improvement in all UKBEC tissues and in 34 out of 42 GTEx GCNs, and the overall improvement is significant (paired t-test *p*-value 2.01e-6).

#### Does a higher enrichment implies less informative modules?

High values of the *S*_*GO*_(*P*) index are of interest, as we prefer a partition *P* over *P* ^′^ if *S*_*GO*_(*P*)>*S*_*GO*_(*P* ^′^). However, it is possible that modules show better *S*_*GO*_ values after k-means because the module have more annotation terms that are generic, and therefore less descriptive about the specifics of the tissue studied. In order to assess this, we applied the notion of information content [31]. We used the GOSim package [32] which applies information-based metrics to Gene Ontology terms. The metric *I* *C*(*t*) for a term *t* belonging to an ontology is defined as:

6 $$ IC(t) = -log P(t),  $$

where *P*(*t*) is the probability of observing *t* within the annotations available within that ontology.

Ideally, we prefer modules with more GO terms, which are more significant (i.e. more reliably defining the network module) and more informative (i.e. terms that are highly specific for the sample’s tissue). From previous sections we know we have more significant networks thanks to *S*_*GO*_. But is k-means capable of not only improving significance but also of maintaining the level of information of the modules if not increasing it?

**Fig. 6:**
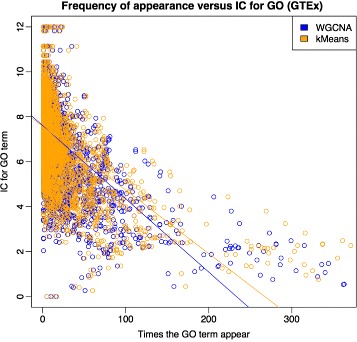
Relation between frequency of appearance of GO annotation terms across all GTEx GCNs and *IC* (information content). Terms appearing more times tend to have lower IC. Regression *lines* show that k-means gets better IC values for highly repetitive terms (not significant Anova test)

Figure 6 displays the differences between standard WGCNA and k-means in the number of times a term appears across all 44 GTEx networks (x-axis) versus their *IC* values (y-axis). Each point represents a significant GO term, obtained by gProfileR as described above. We may expect that terms with lower *IC* values appear more frequently within the GCNs’ functional characterization because they are more abundant on the Gene Ontology. The plot shows that both for kMeans and standard WGCNA there is a clear tendency for the more frequent terms to be also those with lower information content (Pearson correlation −0.58, *p*-value <2.2*e*−16).

Is the overall *IC* obtained by k-means degraded as a consequence of obtaining more significant terms per GCN in comparison with standard WGCNA? To assess this we regressed the information content of the significant annotation terms against the frequency of appearance in the GCN annotation sets. We found a tendency towards higher IC in k-means GCNs. This suggests that k-means annotations are more specific, and therefore more useful.

#### Is the increase in enrichment better than random?

In “Dynamics of k-means when working on 1-TOM distance space” section we noted that one of the changes within cluster partitions after applying k-means is that module sizes change and many modules will increase their size considerably. It is fair to assume that modules increasing their size in genes, will also increase their *s*_*GO*_ enrichment. There is a significant Pearson correlation between increase in module size after k-means and increase in number of significant annotation terms (*r*=0.42, *p*-value 2.2e-16). The question arises, therefore, of what is the real contribution of k-means in comparison to a random shift of genes between modules?

In order to answer this question, for each of the 42 GTEx tissues and their corresponding WGCNA and k-means partitions, we identified those genes that were changed at the WGCNA partition to create the k-means one. Then, in a single step, we randomly assigned these genes to other modules in such a way that we kept the same module sizes obtained with k-means. Via this algorithm, we produced new partitions in which the genes that remained unchanged from WGCNA to k-means stayed at the same modules, but those genes that were changed by k-means were again changed but this time in a random fashion.

**Fig. 7:**
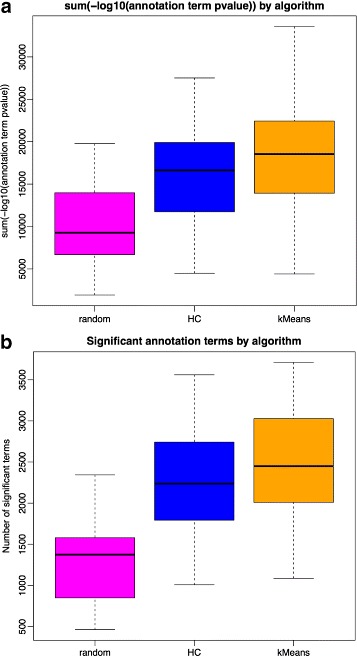
Effect of random assignment of genes selected by k-means, on a WGCNA partition, to be changed from one module to another. Plot (**a**) refers to *S*_*GO*(*P*)_ values and (**b**) to number of significant terms

Figure 7 shows the results of this investigation. Plot (a) shows, for all modules of all GCNs, the *S*_*GO*_(*P*) statistic. Plot (b) shows the number of significant GO terms.

In 89% of the modules, k-means finds the same number (18%) or more (70%) significant GO terms than the random placement of misplaced genes (paired t-test *p*-value <2.2*e*−16). 88% of the final modules show equal (15%) or better (73%) *S*_*GO*_(*P*) index using k-means compared to random (paired t-test *p*-value <2.2*e*−16). Aggregating the results by tissue, k-means placement performs better in all the cases. Interestingly, the random placement of misplaced genes prevents enrichment at the WGCNA partition it starts with (i.e. comparing the magenta with the blue plots). This is important because even though many genes at the WGCNA partition are not touched by the random approach, moving genes randomly will nevertheless worsen these genes’ functional annotations. This suggests that both the number of significant terms and the *S*_*GO*_(*P*) index have a reasonable sensitivity.

### K-means improves brain specific cell type marker enrichment

One interesting property of WGCNA GCNs is that partitions created from them can be useful when studying cell-type specific gene networks. In studies where samples come from bulk tissue, it is most likely that these samples will be comprised of different cell material. In consequence, the gene expression profiles obtained from them should reflect this heterogeneity in some way. WGCNA’s GCNs handle this heterogeneity in an elegant and convenient manner: they often generate gene clusters within partitions which are specialized on a given cell type, i.e. they present a highly significant enrichment of markers (i.e. genes which are differentially expressed) for a given cell type [2, 8, 13, 33].

We wanted to assess the effects of k-means on this particular feature. To do this, we used three different resources defining cell-type specific gene sets. These were WGCNA’s brain lists, [34], and two alternative brain specific sources, labelled here *External* [35] and *Cahoy* [36]. We evaluated each partition’s modules from the 10 UKBEC GCNs and the 13 brain tissue GTEx GCNs, using both standard WGCNA and k-means. This evaluation generated two matrices of *p*-values (i.e. one for WGCNA and one for k-means), with each gene dataset in a row and each specific module from each of the 10 networks in a column. *P*-values reflect a Fisher’s exact test for whether there is significant concentration of the corresponding gene sets in the tested module. We include in Additional files 1 and 2, the results for standard WGCNA and k-means, on the 10 UKBEC GCNs. Note that, in these plots, both columns and rows have been clustered based on −*l* *o* *g*10(*p*-values) so it can be better seen how modules from different tissues cluster together at columns, and also how different gene sets cluster among rows. These heat-maps reveal strongly clustered areas corresponding to groups of cell-type specific genes sets within most, if not all, of the tissues. More specifically, we see four groups cell-type specific gene sets corresponding to microglia, astrocytes, oligodendrocytes and neurons (in order from top right to bottom left).

In the UKBEC k-means heat-map (Additional file 3), using a significance cut-off of 10^−4^ (to account for multiple testing), almost 65% of the modules show cell type enrichment (i.e. 91 modules in total). Within these, 86 modules show a single cell type signal. In the WGCNA heat-map (Additional file 4), 63% of the modules show cell type enrichment (87 modules in total), with 85 showing single cell type signals.

Figure 5 b compares the two enrichment matrices, by aggregating all the cell-type enrichment −*l* *o* *g*_10_ transformed *p*-values as we did for the Gene Ontology enrichment in “K-means improves functional enrichments” section. Each bar represents the sum of all values of the corresponding heat-map, for modules of the given tissue. According to this, we always see an improvement in UKBEC networks and in most of the the GTEx networks. The overall improvement is significant (paired t-test *p*-value 0.000193).

## Conclusions

Our study shows that an additional k-means step, when used as an adjunct to WGCNA, improves the partitions generated from gene co-expression networks. Our method is not an alternative to WGCNA, instead it is an additional step to the standard WGCNA pipeline. Indeed, our method can be applied to any general hierarchical clustering algorithm, and as such it could be usefully applied to any hierarchical clustering based approach for network generation, not just gene co-expression networks.

We evaluated our method using two contrasting gene expression datasets representing a variety of different tissues, one obtained with microarray technology (the UKBEC dataset on 10 brain tissues), and the other with RNA-seq (the GTEx dataset on 42 tissues, which includes 13 brain tissues). Using a variety of approaches, we demonstrate improved performance of our k-means method in both datasets. Furthermore, we also demonstrate improved performance using simulated data generated from the GTEx dataset.

We show via these analyses that it is possible to obtain better partitions for the same networks via our k-means method. Our method generates modules with fewer misplaced genes with respect to their eigengene, and this implies that the eigengene is a better representative of the phenomena hidden behind the particular set of genes belonging to the module.

Using Gene Ontology enrichment analyses, we also show that our partitions are enriched for biological functionality. Statistically significant *S*_*GO*_(*P*) enrichment is seen in all 10 UKBEC CGNs and in 34 out of the 42 GTEx GCNs.

Our partitions have improved modules preservation, which also suggests that the clustering is more accurate from a biological point of view. Although some gene modules are specific of each tissue (and therefore show poor preservation in other tissues), it is a desirable property of most GCN partitions to be highly replicable under the assumption that a preserved module is more likely to be a genuine module. Our analyses suggest that k-means favours the creation of more genuine modules and these results are seen in both UKBEC and GTEx GCNs.

Our k-means method also creates partitions in which gene sets representative of specific brain cell types are seen in modules with increased statistical significance. This suggests, once again, more biologically genuine modules.

GCN construction is likely to become an increasingly important analysis, as genomics and transcriptomics are increasingly applied to aid clinical diagnosis and prognosis. Methods that generate more reliable and robust gene GCNs will enable improved prediction of inter-gene relationships and gene function, with a variety of applications.

## Availability and requirements

UKBEC data [37] has accession code GSE46706. All information about tissues, samples and quality control can be found there. GTEx RPKM gene expression V6 was used in this paper and downloaded from the GTEx portal: http://gtexportal.org/home. Regarding the software we present here, this is the availability and requirements.

**Project name:** km2gcn

**Project homepage:** https://github.com/juanbot/km2gcn

**Operating system:** Linux/Windows/Mac

**Programming language:** R

**Other requirements:** WGCNA R package and gProfileR

**License:** LGPL

## Additional files

Additional file 1 Lists tissues, samples and genes used for the creation of each GCN. (CSV 4 kb)

Additional file 2 Includes comparative results of standard WGCNA and k-means on simulated data. (CSV 4 kb)

Additional file 3 The second one corresponds to k-means. Both on UKBEC datasets. Values higher than 20 are set to 20. Colors at the top of columns correspond to tissues, the tissue legend is at the bottom of columns and cell marker gene set used on the right side. (JPG 1177 kb)

Additional file 4 Heat-maps showing −*l* *o* *g*_10_(*p*-values) from Ficher’s Exact test on significant concentration of specific cell marker gene sets (rows) on each tissue module (columns). The one within the Additional file 1 corresponds to the standard WGCNA. (JPG 1198 kb)

## Abbreviations

BP: Biological process category of the gene ontology
CC: Cellular component category of the gene ontology
EIA: Electronic inferred annotations
GBA: Guilty by association
GCN: Gene co-expression network
GO: Gene ontology
GTEx: Genotype-tissue expression project
HC: Hierarchical clustering
MF: Molecular function category of the gene ontology
MM: Module membership of a gene
SFT: Scale free topology
TOM: Topological overlap matrix
UKBEC: United Kingdom brain expression consortium
WGCNA: Weighted gene-co expression network
