# Supplementary figures and images for: An additional k-means clustering step improves the biological features of WGCNA gene co-expression networks
Source: BMC Syst Biol. 2017 Apr 12;11:47. doi: 10.1186/s12918-017-0420-6 (PMC5389000; doi:10.1186/s12918-017-0420-6)

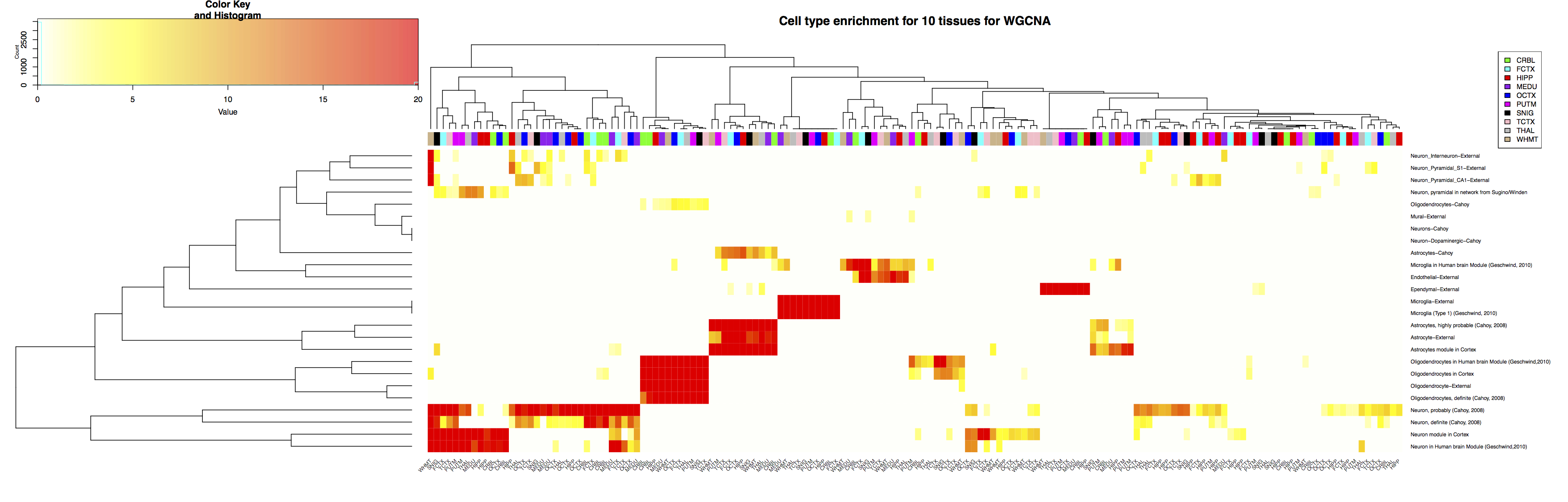

Supplement: Supplementary file 3 — The second one corresponds to k-means. Both on UKBEC datasets. Values higher than 20 are set to 20. Colors at the top of columns correspond to tissues, the tissue legend is at the bottom of columns and cell marker gene set used on the right side. (JPG 1177 kb) [file 12918_2017_420_MOESM3_ESM.jpg]

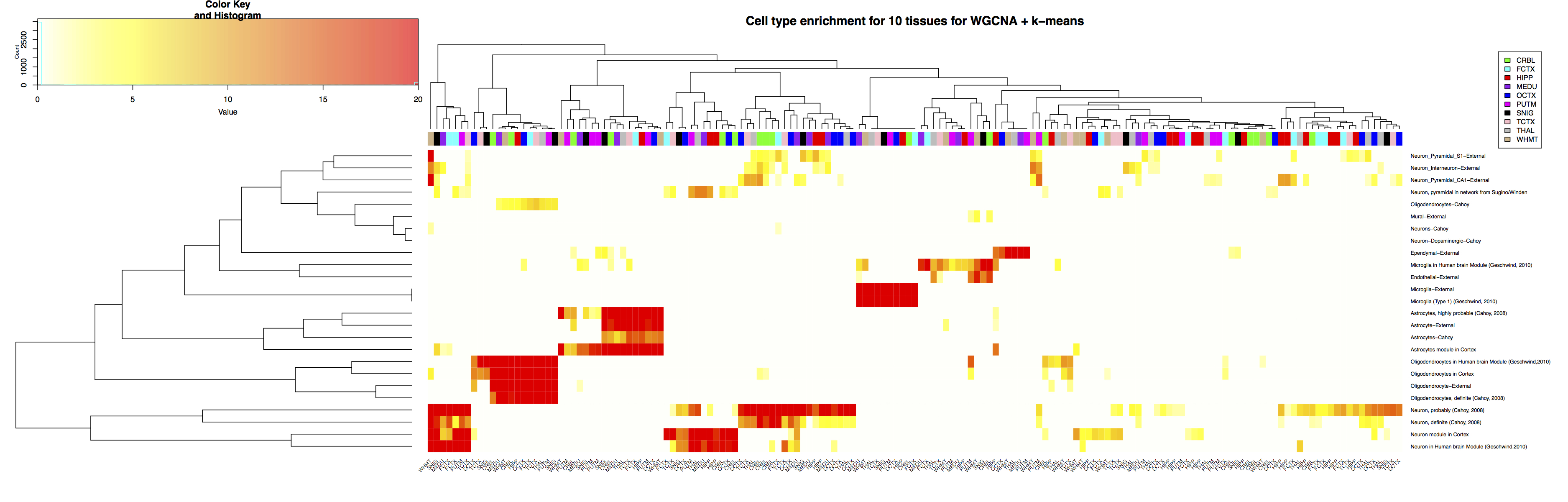

Supplement: Supplementary file 4 — Heat-maps showing −l o g 10(p-values) from Ficher’s Exact test on significant concentration of specific cell marker gene sets (rows) on each tissue module (columns). The one within the Additional file 1 corresponds to the standard WGCNA. (JPG 1198 kb) [file 12918_2017_420_MOESM4_ESM.jpg]
